# Efficacy of adjuvant treatment for fracture nonunion/delayed union: a network meta-analysis of randomized controlled trials

**DOI:** 10.1186/s12891-022-05407-5

**Published:** 2022-05-21

**Authors:** Jun Yang, Xiangmin Zhang, Wangbo Liang, Guo Chen, Yanbo Ma, Yonghua Zhou, Rong Fen, Kaichang Jiang

**Affiliations:** Department of Orthopedics and Traumatology, Yuxi Municipal Hospital of TCM, 53 Nie er Rd, Yuxi, Yunnan Province 653100 People’s Republic of China

**Keywords:** Fracture, Nonunion, Delayed union, Adjuvant treatment, meta-analysis

## Abstract

**Background:**

Fracture nonunion/delayed union seriously affects physical and mental health and quality of life. The aim of this study was to evaluate the relative efficacy of different adjuvant treatments for nonunion/delayed union by network meta-analysis.

**Methods:**

A comprehensive search was performed to identify randomized controlled trials (RCTs) evaluating adjuvant treatment in the management of nonunion/delayed union. A network meta-analysis reporting on healing rate, healing time, and adverse effect (AE) outcomes was conducted to assess and compare different interventions.

**Results:**

Thirty studies were included in the analysis. For the healing rate outcome, bone marrow aspirate (BMA) + autologous cancellous bone (ACB) was found to be significantly better than ACB alone (odds ratio: 0.12; 95% confidence interval: 0.03, 0.59). In the ranking results, BMA+ platelet-rich plasma (PRP) (96%), BMA + ACB (90%), and BMA alone (82%) showed relative advantages in the healing rate. Low-intensity pulsed ultrasonography (LIUS) intervention significantly shortened the healing time compared with ACB (SMD: -9.26; 95% CI: − 14.64, − 3.87). LIUS (100%), BMA + PRP (74%), and bone morphogenetic proteins (BMPs) (69%) have relative advantages. Compared with the control, electromagnetic field (EMF) (OR: 13.21; 95% CI: 1.58, 110.40) and extracorporeal shock wave (ESWT) (OR: 4.90; 95% CI: 1.38, 17.43) had a higher AE risk.

**Conclusions:**

Among the current intervention strategies, BMA in combination with PRP and ACB can improve the healing rate of nonunion/delayed union. LIUS can significantly shorten the healing time. EMF and ESWT may have a high risk of AE. However, large-scale, well-designed studies are still needed to confirm the results.

**Trial registration:**

Retrospectively registered.

**Supplementary Information:**

The online version contains supplementary material available at 10.1186/s12891-022-05407-5.

## Introduction

Fracture nonunion is defined as a fracture that cannot heal in the expected time without further intervention [1]. The clinical manifestations included persistent pain, instability of fracture fragments, and disability. As health care resources, the risk of nonunion/delayed union varies worldwide and is reported to be between 1.9 and 4.9% [2]. Either biological or mechanical factors can contribute to nonunion, seriously affecting physical and mental health and quality of life and increasing health care costs and financial burdens to societies [3].

There is a lack of clinical and radiographic consensus on the standardized definition of nonunion [4, 5]. The US Food and Drug Administration defines nonunion as a fracture that has failed to heal for at least 9 months and has shown no signs of healing for 3 consecutive months [6]. For clinicians, a simplified definition refers to a fracture that will not consolidate without any further intervention, independent of the treatment time [7]. The basic principle of treatment is to provide mechanical and biological support for bone nonunion. Fracture stabilization and immobilization are the primary conditions for treatment [8].

Autologous cancellous bone (ACB) grafts are considered the “gold standard” treatment; however, they are limited by pain at the donor site and potential damage to the arteries, nerves, and tissues around the donor bone [9]. For nonunion, in addition to surgery and ACB transplantation, adjuvant therapy is also a method to improve the healing rate, including physiotherapy, bioactive substance treatment, and oral drugs [10].

In recent meta-analyses, various adjuvant strategies for nonunion were analyzed, in which the application of bone morphogenetic proteins (BMPs) can significantly shorten the healing time, but there are complications of heterotopic ossification [11, 12]. Platelet-rich plasma (PRP) also accelerated nonunion healing, but bisphosphonate had no significant effect [13]. In a network meta-analysis study, low-intensity pulsed ultrasonography (LIUS) was considered to have more advantages than electrical stimulation in the healing effect of fresh fractures [14]. The aim of this study was to evaluate the relative efficacy of different adjuvant treatments for nonunion/delayed union by network meta-analysis.

## Methods

This meta-analysis followed the Cochrane handbook for conducting and the Preferred Reporting Items for Systematic Reviews and Meta-Analyses (PRISMA) guidelines for reporting. Two authors separately conducted literature retrieval, study eligibility, data extraction, and quality assessment with inconsistency solved by discussion and decided by the corresponding author.

### Literature search

A literature search of PubMed, Embase, Cochrane, Ebscohost, and Scopus was performed from databases incipient to November 2021 for randomized controlled trials (RCTs) evaluating adjuvant treatment in the management of nonunion/delayed union. The brief retrieval formula was “(fracture) AND random* AND ((((Non-Union) OR (Delayed Union)) OR (Mal-Union)) OR (nonunion)) OR (nonunions))” (details in the supplementary file). The references of related reviews were also screened to prevent omissions.

### Inclusion and exclusion criteria

We included RCTs evaluating adjuvant therapy for patients with nonunion or delayed union. The inclusion criteria were as follows: 1, the study was an RCT design; 2, nonunion/delayed union patients were analyzed; 3, the intervention was adjuvant therapy; 4, the control was another adjuvant therapy or blank control that was different from the intervention; and 5, the outcome reported fracture healing rate and/or fracture healing time. The exclusion criteria were as follows: 1, intervention studies on fresh fractures; 2, animal and experimental studies; and 3, studies that did not report the predetermined outcomes. Furthermore, reviews, case reports, conference abstracts, editorials, and comments were also excluded.

### Data extraction and quality assessment

The contents were extracted by two authors, and disagreements were resolved by consensus. The following data were extracted: the first author’s name, publication year, study location, sample size, patients’ average age, fracture site, clinical diagnosis, intervention, control and follow-up. The outcomes were radiographic/clinical healing rate, healing time, and adverse effects (AEs). AEs defined as all medical complications, adverse events, and reoperations reported from each included study. The methodological quality of the eligible studies was evaluated with the Cochrane risk of bias tool.

### Statistical analysis

Discontinuous variables were pooled using odds ratios (ORs) with 95% confidence intervals (CIs), and continuous variables were pooled using standard mean differences (SMDs) with 95% CIs. A random-effects network meta-analysis with a frequentist framework was adopted. Network plots were generated for each outcome, with nodes representing the type of therapeutic strategy, lines between nodes representing direct comparison, and edges of lines representing the precision of comparison. Inconsistency was locally assessed by calculating the difference between direct and indirect estimates in network analysis. Heterogeneity analysis of direct comparisons was performed. For each outcome, we estimated the ranking probability of the therapeutic strategy being at each possible rank for each therapeutic strategy using a surface under the cumulative ranking curve (SUCRA). Comparison-adjusted funnel plots were used to determine the possible publication bias. Subgroup analyses were also performed according to long bone and short bone nonunion. *p* < 0.05 was considered a significant difference, and data were analyzed by R language (version 4.0.3) with the “netmeta” package (version 2.1–0). The R code is shown in the supplementary file.

## Results

### Literature search

**Fig. 1:**
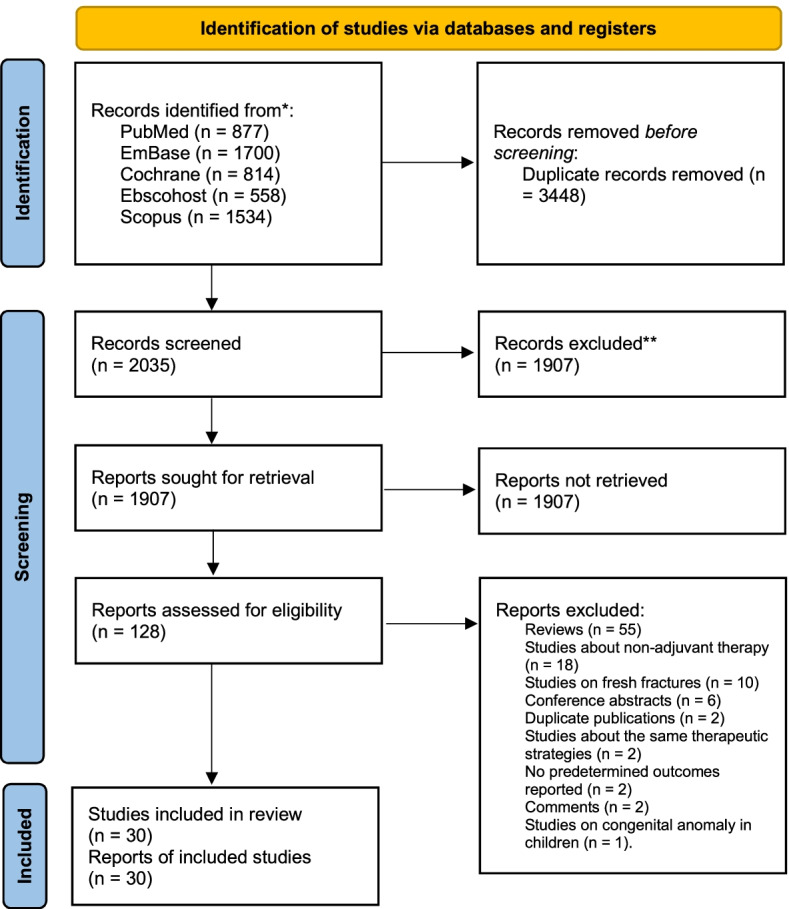
Flowchart illustrating the literature search and the selection of included studies

In this study, 2035 articles were identified after duplications were removed. After screening the titles and abstracts, 1907 articles were excluded. The full texts of the remaining 128 articles were assessed. The following articles were excluded: reviews (55); studies about nonadjuvant therapy (18); studies on fresh fractures (10); conference abstracts (6); duplicate publications (2); studies about the same therapeutic strategies (2); no predetermined outcomes reported (2); comments (2); and studies on congenital anomaly in children (1). Finally, 30 studies were included in the analysis [15–44] (Fig. 1).

### Characteristics of the included studies

**Fig. 2:**
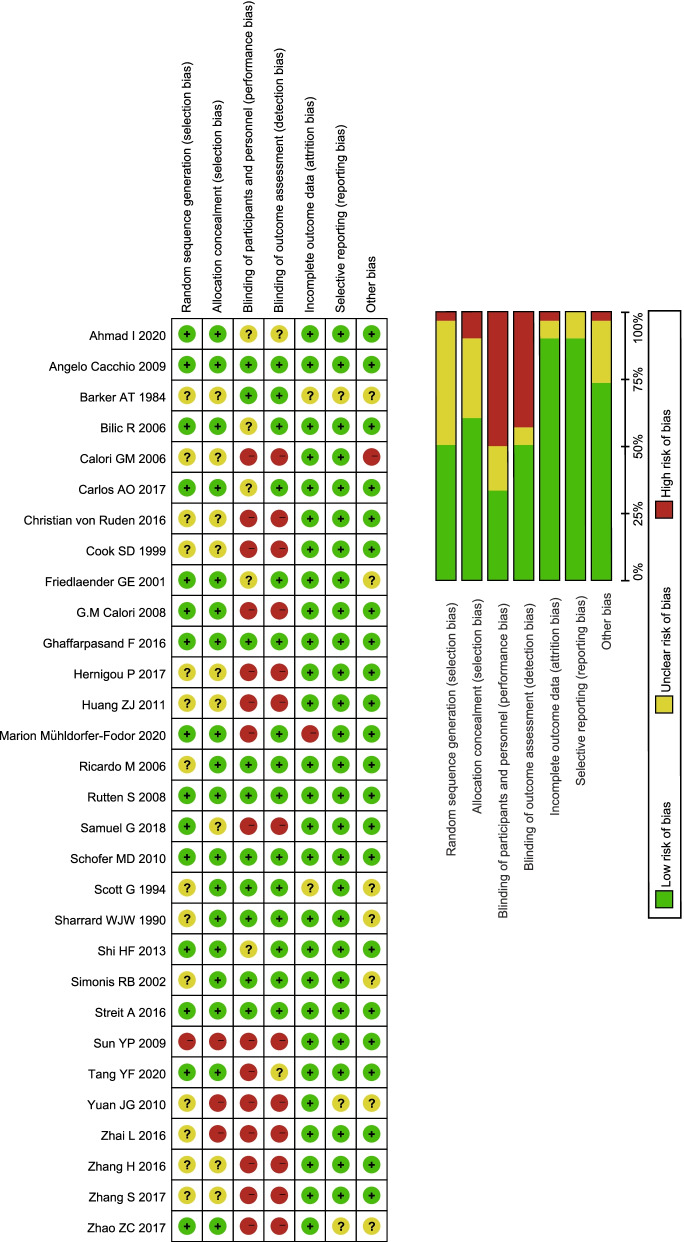
Risk of bias graph for each included study

The included studies were published from 1984 to 2020. The research is mainly located in Asia, North America, and Europe. The sample size from each study was relatively small, with a maximum of 140 patients. One study included adolescent patients [17]; however, we do not think it will impact the overall results. The interventions analyzed included physiotherapy (LIUS, extracorporeal shock wave (ESWT), electromagnetic field (EMF) therapy), bioactive substance treatment (PRP, BMP, bone marrow aspirate (BMA, including mesenchymal stem cells)), and oral drugs (strontium ranelate (Protelos), Chinese traditional medicine (CTM)). The average follow-up period ranged from 3 months to 7.6 years (Table 1). After comprehensive consideration of the risk of bias, although all studies were RCTs, the major factor affecting the quality was that patients, intervention personnel, and the outcome assessor were not blinded, which may lead to a subjective influence on the healing judgment, giving the results a positive trend (Fig. 2).

**Table 1:** Characteristics of the included studies

Study	Location	Sample size	Average age	Fracture site	Interventions	Control	Follow-up
Tang YF 2020 [15]	China	66	20–56	Tibia	PRP + BMA	BMA	16–36 Months
Marion Mühldorfer-Fodor 2020 [16]	Germany	68	29(16–60)	Scaphoid	EWST+ACB	ACB	24 Weeks
Ahmad I 2020 [17]	Pakistan	101	31.46 ± 13.9	Tibia (major)	PROTELOS	CONTROL	3 Months
Samuel G 2018 [18]	Indian	40	34.3 ± 9.49;39.35 ± 10.39	Tibia (major)	PRP	CONTROL	36 Weeks
Carlos AO 2017 [19]	Germany	16	37.14 ± 10.22;38.88 ± 15.36	Humerus	PRP + ACB	ACB	36 Weeks
Hernigou P 2017 [20]	France	80	41.2 ± 18.2	Tibia	BMA + ACB	ACB	7.6 (5–10) Years
Zhang S 2017 [21]	China	47	38.29 ± 7.49;36.57 ± 8.31	Tibia (major)	PRP + BMA	BMA	9–15 Months
Zhao ZC 2017 [22]	China	92	18–70	Femur	PRP	CONTROL	12 Months
Ghaffarpasand F 2016 [23]	Iran	75	26.5 ± 5.8;26.3 ± 6.2	Femur, Tibia	PRP	CONTROL	12 Months
Zhang H 2016 [24]	China	24	33.45;32.69	Tibia	BMA	CONTROL	12–34 Months
Streit A 2016 [25]	USA	8	47(24–63)	Fifth metatarsal	EMF	CONTROL	24 Weeks
Christian von Ruden 2016 [26]	Germany	49	44(19–77)	Ulnar, Radial	BMP + ACB	ACB	6 Months
Zhai L 2016 [27]	China	63	39.6(23–50);38.1(20–49)	Femur, Tibia	BMA + ESWT	ESWT	12 Months
Shi HF 2013 [28]	China	58	41.1 ± 14.5;38.4 ± 11.6	Femur, Tibia	EMF	CONTROL	3 Months
Huang ZJ 2011 [29]	China	64	47.3 ± 22.2	Ulna or radius (major)	CTM	CONTROL	3 Months
Schofer MD 2010 [30]	USA	101	42.6 ± 14.6;45.1 ± 11.9	Tibia	LIUS	CONTROL	16 Weeks
Yuan JG 2010 [31]	China	140	34 ± 2	Humerus, Tibia	BMA	ACB	3 Months
Sun YP 2009 [32]	China	40	43 ± 6	Tibia (major)	BMP + ACB	ACB	11–20 Months
Angelo Cacchio 2009 [33]	Italy	126	42.7 ± 5.9	Tibia Femur	ESWT	CONTROL	24 Months
Rutten S 2008 [34]	Netherlands	13	42–63	Fibula	LIUS	CONTROL	3 Months
G.M Calori 2008 [35]	Italy	120	43(19–65)	Humerus, Tibia (major)	BMP	PRP	9 Months
Bilic R 2006 [36]	Croatia	18	23 ± 5;22 ± 5;19 ± 4	Scaphoid	BMP + ACB	ACB	24 Months
Ricardo M 2006 [37]	France	21	26.7(17–42)	Scaphoid	LIUS	CONTROL	1–4 Years
Calori GM 2006 [38]	Italy	29	47 ± 2.56;35.3 ± 1.76	Femur, Tibia	BMP	PRP	9 Months
Simonis RB 2002 [39]	UK	34	32(16–61)	Tibia	EMF	CONTROL	6 Months
Friedlaender GE 2001 [40]	USA	122	38 ± 16;34 ± 11	Tibia	BMP + ACB	ACB	24 Months
Cook SD 1999 [41]	USA	30	na	Tibia	BMP	ACB	9 Months
Scott G 1994 [42]	UK	23	43(23–87)	Femur, Tibia	EMF	CONTROL	6 Months
Sharrard WJW 1990 [43]	**UK**	**51**	**34.7(18–84);45.4(18–76)**	**Tibia**	EMF	**CONTROL**	**12 Weeks**
Barker AT 1984 [44]	UK	17	34(19–72)	Tibia	EMF	CONTROL	48 Weeks

*Abbreviations*: *ACB* autologous cancellous bone, *BMA* bone marrow aspirate, *BMP* bone morphogenetic protein, *CTM* Chinese traditional medicine, *EMF* electromagnetic field, *ESWT* extracorporeal shock wave, *LIUS* low-intensity pulsed ultrasonography, *PROTELOS* strontium ranelate, *PRP* platelet-rich plasma

In the classification of adjuvant therapeutic strategies, surgical stabilization was necessary for long bone nonunion treatment but not for short bones. Therefore, surgical fixation was adopted according to actual needs and was not considered in the strategies. However, ACB, as the gold standard treatment, might affect adjuvant therapy and is considered a strategy. Additionally, in the healing rate outcome, both intervention groups might achieve fracture healing during the follow-up period. In this case, the healing time is an important supplement to assess adjuvant therapy effects.

### Results of network meta-analysis

**Fig. 3:**
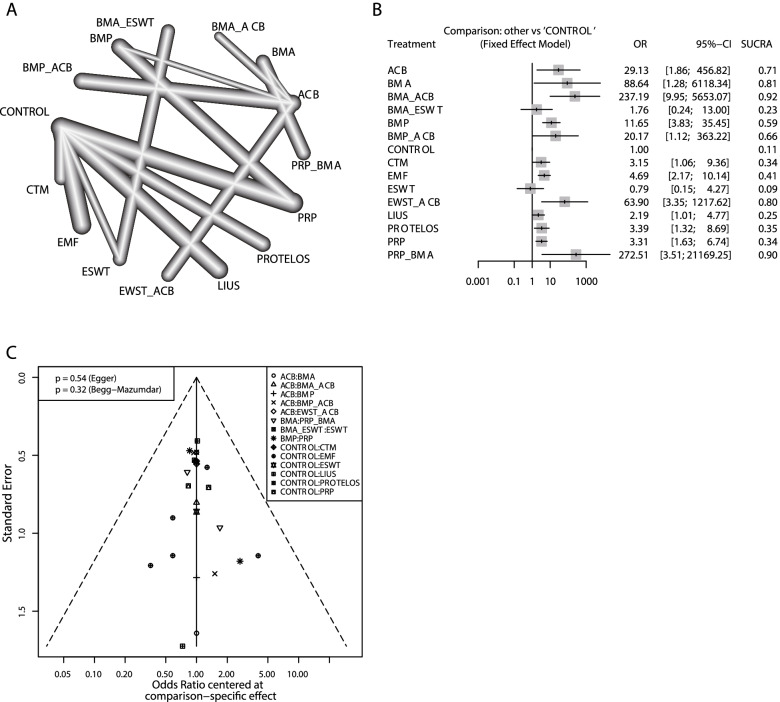
The network meta-analysis results on healing rate. **A** Network plot; **B** Forest plot; **C** Funnel plot. ACB: autologous cancellous bone; BMA: bone marrow aspirate; BMP: bone morphogenetic proteins; CTM: Chinese traditional medicine; EMF: electromagnetic fields; ESWT: extracorporeal shock wave; LIUS: low-intensity pulsed ultrasonography; PROTELOS = strontium ranelate; PRP: platelet-rich plasma

For healing rate outcome, 15 intervention strategies, from 30 studies with 1711 patients were analyzed, including ACB, BMA, BMP, CTM, EMF, ESWT, LIUS, PROTELOS, PRP, their combinations, and blank control (Fig. 3A). In pairwise comparisons, only ESWT (OR: 1.76; 95% CI: 0.24, 13.00) and BMA + ESWT (OR: 0.79; 95% CI: 0.15, 4.27) were not significantly different from the blank control. Compared to ACB, only BMA + ACB was significantly better (OR: 0.12; 95% CI: 0.03, 0.59). The heterogeneity analysis of direct comparisons and league tables is shown in the Supplementary file. In the SUCRA ranking results, BMA + PRP (96%), BMA + ACB (90%), and BMA alone (82%) showed relative advantages in healing rate (Fig. 3B). A comparison-adjusted funnel plot did not show potential publication bias (Fig. 3C). In the long bone subgroup results, BMA + ACB (SUCRA: 0.92) and PRP + BMA(SUCRA:0.91) have a relative advantage. In the short bone subgroup, only one study reported a comparison between EWST+ACB and ACB without a significant difference [16]. In the remaining studies, all patients recovered (detailed in the Supplementary file).

**Fig. 4:**
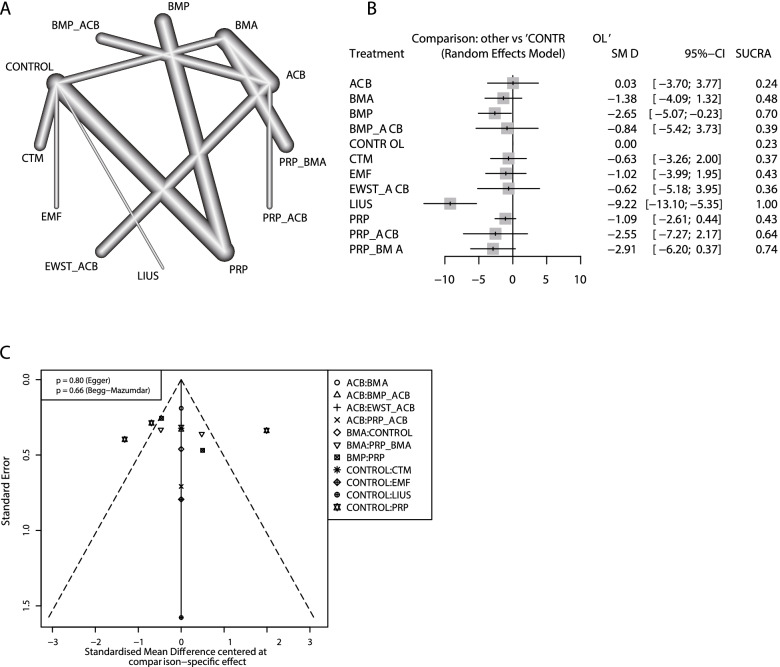
The network meta-analysis results on healing time. **A** Network plot; **B** Forest plot; **C** Funnel plot. ACB: autologous cancellous bone; BMA: bone marrow aspirate; BMP: bone morphogenetic proteins; CI: confidence interval; CTM: Chinese traditional medicine; EMF: electromagnetic fields; ESWT: extracorporeal shock wave; LIUS: low-intensity pulsed ultrasonography; OR: odds ratio; PROTELOS = strontium ranelate; PRP: platelet-rich plasma; SUCRA: surface under the cumulative ranking curve

For the healing time, a total of 13 intervention strategies from 15 studies with 702 patients were analyzed, including ACB, BMA, BMP, CTM, EMF, EWST, LIUS, PRP, their combination, and blank control (Fig. 4A). In the pairwise comparison, BMP (SMD: -2.65; 95% CI: − 5.07, − 0.23) and LIUS (SMD: -9.22; 95% CI: − 13.10, − 5.35) interventions had significantly shorter healing times than the blank control. Compared with ACB, only LIUS intervention significantly shortened the healing time (SMD: -9.26; 95% CI: − 14.64, − 3.87) (detailed in the Supplementary file). In the SUCRA ranking results, LIUS (100%), PRP + BMA (74%), and BMP (69%) had relative advantages (Fig. 4B). No publication bias was detected (Fig. 4C). In the long bone subgroup, PRP + BMA (SUCRA: 0.81) had a relative advantage. In the short bone subgroup, LIUS (SUCRA: 1.00) has advantages (details in the Supplementary file).

**Fig. 5:**
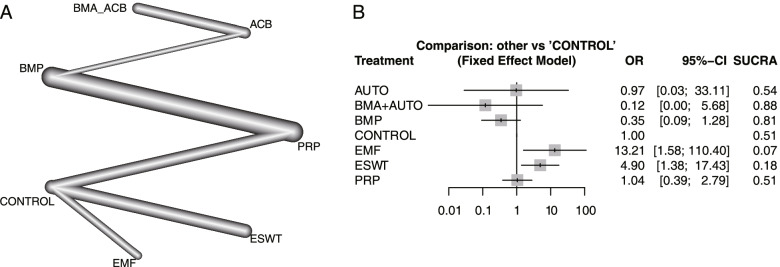
The network meta-analysis results on AE. **A** Network plot; **B** Forest plot. ACB: autologous cancellous bone; BMA: bone marrow aspirate; BMP: bone morphogenetic protein; EMF: electromagnetic field; ESWT: extracorporeal shock wave; PRP: platelet-rich plasma

For AE outcome, a total of 7 intervention strategies from 10 studies with 799 patients were analyzed, including ACB, BMA, BMA + ACB, BMP, EMF, ESWT, PRP, and control (Fig. 5A). Compared with the control, EMF (OR: 13.21; 95% CI: 1.58, 110.40) and ESWT (OR: 4.90; 95% CI: 1.38, 17.43) had a higher AE risk. In the SUCRA ranking results, BMA + ACB (95%) had relatively few side effects (Fig. 5B). Publication bias analysis was not performed due to fewer than 10 included studies. AE results were all based on long bone nonunion. Therefore, subgroup analysis was not performed. For infection-related AE results, ESWT had a relatively low risk (SUCRA: 0.86), but it was not significantly different from the blank control. The AE results included malunion, infection, reoperation, and adverse drug reactions with high heterogeneity. AE items and infection-related AE results are shown in the Supplementary file.

## Discussion

In this study, adjuvant treatments for fracture nonunion/delayed union were comprehensively analyzed by network meta-analysis. Adjuvant therapeutic strategies include ACB, BMA, BMP, CTM, EMF, ESWT, LIUS, PROTELOS, PRP, and their combinations. In the healing rate results, BMA + PRP, BMA + ACB, and BMA have relative advantages that support BMA application in nonunion/delayed union treatment. The healing time results showed that LIUS and BMP, especially LIUS, could significantly shorten the healing time. In the AE results, EMF and ESWT interventions may have a high risk.

BMA harvested from bone marrow includes multiple types of progenitor cells, and is injected into the fracture site, which is considered a useful treatment for bone regeneration. Centrifugal concentration is beneficial to improve the effect [45]. Especially for atrophic nonunion, the proliferation and osteogenic differentiation ability of mesenchymal stem cells were inhibited, and BMA supplied more active mesenchymal stem cells [46]. Generally, long bone nonunion treatment requires surgical intervention, and BMA is easier to obtain and apply locally. Therefore, compared to other cell therapy-related treatments, this method has the advantage of convenience. BMA mainly has three application strategies, including intraoperative injection, transplantation after in vitro expansion, and soaked ACB or other bone graft materials [47, 48]. However, osteofascial compartment syndrome after multiple injections should be especially considered. In addition, BMA + PRP also achieved ideal effects. PRP contains active cytokine components, such as platelet-derived growth factor and vascular endothelial growth factor, which promote osteogenic-angiogenic coupling in bone formation with BMA [49].

LIUS is a physical intervention approved by the FDA for nonunion treatment [50]. As extracorporeal equipment, pressure waves are delivered transcutaneously to the fracture site with slight heat. Its mechanism may vary, including the promotion of bone regeneration, angiogenesis, and nerve regeneration [51, 52]. The results of this study also suggested that it may be the only intervention to shorten the healing time of nonunion/delayed union compared with ACB.

BMPs are the most widely used growth factors in bone nonunion. BMP2 and BMP7 have been approved for clinical use for repairing long bone nonunion fractures [53]. This method is successful in new bone formation, but heterotopic ossification is also a major problem in clinical application. In this study, BMPs significantly shortened the healing time without increasing the AE risk. However, supraphysiological dose application and the lack of a controlled delivery system can lead to heterotopic ossification that requires well-planned surgical excision [54–56]. This serious AE still raises concern in clinical applications.

In the field of nonunion, some groups have made important contributions. Giannoudis and his team are widely known for the “diamond concept” (the basic therapeutic concept of bone healing). Their recent study comprehensively analyzed the biological, molecular, and genetic profiles associated with fracture nonunion that provide evidence for macroscopic appearances of nonunion and potential biomarkers in clinical practice [57]. In the clinic, they constructed a nonunion risk model that reliably identified high-risk patients early [58]. Schmidmaier and his group further explained the application of the “diamond concept” in personalized treatment strategies [59]. They also described the effect of occult infection on fracture recovery after autologous bone transplantation [60], and contrast-enhanced ultrasonography can be an appropriate diagnostic tool for predicting long bone nonunion [61]. Calori’s group described a clinical protocol for the treatment of posttraumatic septic bone defects using a giant prosthesis and reconstruction of the patellar tendon [62].

In a previous meta-analysis of adjuvant treatment of bone nonunion, LIUS could be used as an alternative treatment for surgery, especially for patients whose surgery is high risk [63]. Our results suggested that LIUS can further shorten the healing time. In another meta-analysis, the application of BMPs achieved a healing rate similar to autogenous bone transplantation [11]. Our study concluded that there is still more advantageous adjuvant therapy than BMP and autogenous bone transplantation.

The present study has several limitations. First, this study was performed at the study level, instead of at the individual level, and hypertrophic nonunion and atrophic nonunion patients could not be analyzed in subgroups. Second, there was no limited place at the time of study publication, but a long time span (nearly 40 years) may lead to considerable changes in medical conditions. Third, radiographic healing was adopted as a priority, and clinical healing was also adopted if radiographic healing was not reported. Fourth, the impact of drug dosage or electromagnetic intensity on fracture healing was not assessed due to the small number of eligible studies.

In summary, this study showed that among the current intervention strategies, BMA + PRP, BMA + ACB, and BMA have relative advantages in improving the healing rate that supports BMA application in nonunion/delayed union treatment. LIUS and BMP, especially LIUS, can significantly shorten the healing time. EMF and ESWT interventions may bring a high risk of AEs. However, large-scale, well-designed studies are still needed to confirm the results.

## Supplementary Information

**Additional file 1.**

## Data Availability

All data generated or analyzed during this study are presented in this published article and its additional information files. Meta-analysis data can be requested from corresponding author.

## Abbreviations

ACB: Autologous cancellous bone
AE: Adverse effect
BMA: Bone marrow aspirate
BMPs: Bone morphogenetic proteins
CTM: Chinese traditional medicine
EMF: Electromagnetic field
ESWT: Extracorporeal shock wave
LIUS: Low-intensity pulsed ultrasonography
OR: Odds ratio
PRISMA: Preferred Reporting Items for Systematic Reviews and Meta-Analyses
PRP: Platelet-rich plasma
RCTs: Randomized controlled trials
SMD: Standardized Mean difference
SUCRA: Surface under the cumulative ranking curve
