# Supplementary material for: Efficacy of adjuvant treatment for fracture nonunion/delayed union: a network meta-analysis of randomized controlled trials
Source: BMC Musculoskelet Disord. 2022 May 21;23:481. doi: 10.1186/s12891-022-05407-5 (PMC9123731; doi:10.1186/s12891-022-05407-5)

Supplementary file

[The detailed search strategy in the PubMed database 2](#_Toc100308939)

[The R code used in this network meta-analysis 3](#_Toc100308940)

[The heterogeneity results of direct comparisons. 7](#_Toc100308941)

[League tables 9](#_Toc100308942)

[Subgroup analysis 12](#_Toc100308943)

[AE items of each reported study and infection related AE results 17](#_Toc100308944)

# The detailed search strategy in the PubMed database

| Search number | Query | Search Details | Results |
| --- | --- | --- | --- |
| 4 | ((#1) AND (#2)) AND (#3) | ("Non-Union"[All Fields] OR (("delay"[All Fields] OR "delayed"[All Fields] OR "delaying"[All Fields] OR "delays"[All Fields]) AND ("union"[All Fields] OR "union s"[All Fields] OR "unionism"[All Fields] OR "unionization"[All Fields] OR "unionize"[All Fields] OR "unionizing"[All Fields] OR "unions"[All Fields])) OR "Mal-Union"[All Fields] OR ("nonunion"[All Fields] OR "nonunions"[All Fields]) OR ("nonunion"[All Fields] OR "nonunions"[All Fields])) AND ("fractur"[All Fields] OR "fractural"[All Fields] OR "fracture s"[All Fields] OR "fractures, bone"[MeSH Terms] OR ("fractures"[All Fields] AND "bone"[All Fields]) OR "bone fractures"[All Fields] OR "fracture"[All Fields] OR "fractured"[All Fields] OR "fractures"[All Fields] OR "fracturing"[All Fields]) AND "random*"[All Fields] | 877 |
| 3 | random* | "random*"[All Fields] | 1,549,547 |
| 2 | fracture | "fractur"[All Fields] OR "fractural"[All Fields] OR "fracture s"[All Fields] OR "fractures, bone"[MeSH Terms] OR ("fractures"[All Fields] AND "bone"[All Fields]) OR "bone fractures"[All Fields] OR "fracture"[All Fields] OR "fractured"[All Fields] OR "fractures"[All Fields] OR "fracturing"[All Fields] | 343,250 |
| 1 | ((((Non-Union) OR (Delayed Union)) OR (Mal-Union))  OR (nonunion)) OR (nonunions) | "Non-Union"[All Fields] OR (("delay"[All Fields] OR "delayed"[All Fields] OR "delaying"[All Fields] OR "delays"[All Fields]) AND ("union"[All Fields] OR "union s"[All Fields] OR "unionism"[All Fields] OR "unionization"[All Fields] OR "unionize"[All Fields] OR "unionizing"[All Fields] OR "unions"[All Fields])) OR "Mal-Union"[All Fields] OR ("nonunion"[All Fields] OR "nonunions"[All Fields]) OR ("nonunion"[All Fields] OR "nonunions"[All Fields]) | 21,687 |

# The R code used in this network meta-analysis

#For healing rate calculation

#R version 4.1.2

#meta version 5.2-0

#netmeta version 2.1-0

library(netmeta)

library(readxl)

library(WriteXLS)

library(nmarank)

#import data from clipboard

#For binary outcome, title: studlab treat1 event1 n1 treat2 event2 n2

#For continuous outcomes, title: studlab treat1 n1 y1 sd1 treat2 n2 y2 sd2

net1 <- read.delim("clipboard",header = T)

#import data from csv file

net1 <- read.csv("healing rate.csv",header = T)

#For binary outcome data, small.values set whether small treatment effects indicate a beneficial ("good") or harmful ("bad") effect

#For healing rate:small.values = "bad"

#For Adverse Effect: small.values = "good"

p1 <- pairwise(list(treat1, treat2),

n = list(n1, n2),

event = list(event1, event2),

data = net1, studlab = studlab, sm="OR")

net2 <- netmeta(p1, reference.group = "Control",small.values = "bad")

#For continuous outcomes data

p1 <- pairwise(list(treat1, treat2),

n = list(n1, n2),

mean = list(y1, y2),

sd = list(sd1, sd2),

data = net1, studlab =studlab, sm='SMD')

net2 <- netmeta(p1, reference.group = "Control",small.values = "good")

#Draw forest plot

forest(net2, rightcols = c("effect", "ci", "SUCRA"),

just.addcols = "right")

#Draw network plot

netgraph(net2)

#Ranking according to P-score

rank1 <- netrank(net2)

#Ranking according to SUCRA

rank1 <- netrank(net2, method = "SUCRA", nsim = 100)

#Draw split network plot

ns1 <- netsplit(net2)

forest(ns1, fontsize = 8, spacing = 0.5, addrow.subgroups = FALSE,

show = "with.direct", sm="OR")

#League table

nl1 <- netleague(net2, comb.fixed = T, comb.random = T, digits = 2, bracket = "(", separator = " - ")

write.table(nl1$fixed, file = "league0-fixed.csv",

row.names = FALSE, col.names = FALSE,

sep = ",")

write.table(nl1$random, file = "league0-random.csv",

row.names = FALSE, col.names = FALSE,

sep = ",")

#Conduct pairwise meta-analyses for all comparisons with direct evidence

np1 <- netpairwise(net2)

print(np1)

#Draw funnel plot

ord <- net2$trts

funnel(net2, order = ord,

method.bias = c("Egger","Begg"), digits.pval = 2)

# The heterogeneity results of direct comparisons.

Table 1. **Pairwise meta-analyses of all directly compared interventions with statistical heterogeneity on healing rate outcome.**

| Comparisons | Number of studies | OR | 95%CI | Q | I^2^ |
| --- | --- | --- | --- | --- | --- |
| CTM:CONTROL | 1 | 3.15 | [1.06; 9.36] | 0 | -- |
| PRP:CONTROL | 3 | 3.31 | [1.63; 6.74] | 0.23 | 0 |
| EMF:CONTROL | 5 | 4.69 | [2.17; 10.14] | 2.95 | 0 |
| BMA:PRP_BMA | 2 | 0.33 | [0.12; 0.89] | 0.42 | 0 |
| ACB:BMA_ACB | 1 | 0.12 | [0.03; 0.59] | 0 | -- |
| LIUS:CONTROL | 2 | 2.19 | [1.01; 4.77] | 0.04 | 0 |
| ACB:EWST_ACB | 1 | 0.46 | [0.16; 1.31] | 0 | -- |
| PROTELOS:CONTROL | 1 | 3.39 | [1.32; 8.69] | 0 | -- |
| ACB:BMP_ACB | 2 | 1.44 | [0.60; 3.50] | 0.12 | 0 |
| ACB:BMA | 1 | 0.33 | [0.01; 8.21] | 0 | -- |
| ESWT:CONTROL | 1 | 0.79 | [0.15; 4.27] | 0 | -- |
| BMP:PRP | 2 | 3.52 | [1.49; 8.29] | 0.8 | 0 |
| ACB:BMP | 1 | 2.5 | [0.20; 31.00] | 0 | -- |
| BMA_ESWT:ESWT | 1 | 2.24 | [0.77; 6.49] | 0 | -- |

Table 2. **Pairwise meta-analyses of all directly compared interventions with statistical heterogeneity on healing time outcome.**

| Comparisons | Number of studies | SMD | 95%-CI | Q | I^2^ |
| --- | --- | --- | --- | --- | --- |
| CTM:CONTROL | 1 | -0.63 | [ -1.26; 0.00] | 0 | -- |
| PRP:CONTROL | 3 | -1.12 | [ -1.50; -0.75] | 53.37 | 96.30% |
| ACB:PRP_ACB | 1 | 2.58 | [ 1.25; 3.92] | 0 | -- |
| BMA:PRP_BMA | 2 | 1.49 | [ 1.02; 1.97] | 3.94 | 74.60% |
| LIUS:CONTROL | 1 | -9.22 | [-12.14; -6.30] | 0 | -- |
| BMA:CONTROL | 1 | -1.38 | [ -2.28; -0.49] | 0 | -- |
| ACB:EWST_ACB | 1 | 0.65 | [ 0.06; 1.24] | 0 | -- |
| EMF:CONTROL | 1 | -1.02 | [ -2.54; 0.50] | 0 | -- |
| ACB:BMA | 1 | 1.42 | [ 1.04; 1.79] | 0 | -- |
| ACB:BMP_ACB | 1 | 0.88 | [ 0.23; 1.53] | 0 | -- |
| BMP:PRP | 2 | -1.79 | [ -2.23; -1.35] | 3.09 | 67.70% |

Table 3. **Pairwise meta-analyses of all directly compared interventions with statistical heterogeneity on adverse effect outcome.**

| Comparisons | Number of studies | OR | 95%-CI | Q | I^2^ |
| --- | --- | --- | --- | --- | --- |
| PRP:CONTROL | 2 | 1.04 | [0.39; 2.79] | 0.44 | 0.00% |
| AUTO:BMA+AUTO | 1 | 8.14 | [1.69; 39.32] | 0 | -- |
| EMF:CONTROL | 2 | 13.21 | [1.58; 110.40] | 0.34 | 0.00% |
| ESWT:CONTROL | 1 | 4.9 | [1.38; 17.43] | 0 | -- |
| BMP:PRP | 2 | 0.33 | [0.14; 0.79] | 0 | 0.00% |
| AUTO:BMP | 1 | 2.81 | [0.11; 74.56] | 0 | -- |

# League tables

Table 1. Pairwise comparisons of the relative effects of interventions on healing rate.

| **ACB** | 0.33  (0.01, 8.21) | **0.12**  **(0.03, 0.59)** | . | 2.50  (0.20, 31) | 1.44  (0.60, 3.50) | . | . | . | . | 0.46 (0.16, 1.31) | . | . | . | . |
| --- | --- | --- | --- | --- | --- | --- | --- | --- | --- | --- | --- | --- | --- | --- |
| 0.33  (0.01, 8.21) | **BMA** | . | . | . | . | . | . | . | . | . | . | . | . | **0.33 (0.12, 0.89)** |
| 0.12  (0.03, 0.59) | 0.37  (0.01, 13.44) | **BMA_ACB** | . | . | . | . | . | . | . | . | . | . | . | . |
| 16.52  (0.55, 495) | 50.27  (0.47, 5428) | 134.51  (3.17, 5707) | **BMA_ESWT** | . | . | . | . | . | 2.24  (0.77, 6.49) | . | . | . | . | . |
| 2.50  (0.20, 31) | 7.61  (0.13, 453) | 20.36  (1.04, 397) | 0.15  (0.02, 1.49) | **BMP** | . | . | . | . | . | . | . | . | **3.52**  **(1.49, 8.29)** | . |
| 1.44  (0.60, 3.50) | 4.40  (0.16, 124) | 11.76  (1.93, 71.56) | 0.09  (0.00, 2.94) | 0.58  (0.04, 8.33) | **BMP_ACB** | . | . | . | . | . | . | . | . | . |
| 29.13  (1.86, 457) | 88.64  (1.28, 6118) | 237.19  (9.95, 5653) | 1.76  (0.24, 13.00) | 11.65  (3.83, 35.45) | 20.17  (1.12, 363) | **CONTROL** | **0.32**  **(0.11, 0.94)** | **0.21**  **(0.10, 0.46)** | 1.27  (0.23, 6.87) | . | **0.46**  **(0.21, 0.99)** | **0.30**  **(0.12, 0.76)** | **0.30**  **(0.15, 0.61)** | . |
| 9.24  (0.48, 178.) | 28.13  (0.36, 2228) | 75.27  (2.63, 2151) | 0.56  (0.06, 5.44) | 3.70  (0.78, 17.53) | 6.40  (0.29, 141) | 0.32  (0.11, 0.94) | **CTM** | . | . | . | . | . | . | . |
| 6.22  (0.36, 108) | 18.92  (0.26, 1400) | 50.62  (1.94, 1323) | 0.38  (0.04, 3.20) | 2.49  (0.64, 9.63) | 4.30  (0.22, 85.77) | 0.21  (0.10, 0.46) | 0.67  (0.18, 2.55) | **EMF** | . | . | . | . | . | . |
| 36.94  (1.46, 934) | 112.41  (1.18, 10734) | 300.79  (8.28, 10932) | 2.24  (0.77, 6.49) | 14.78  (1.95, 112) | 25.57  (0.90, 728) | 1.27  (0.23, 6.87) | 4.00  (0.54, 29.82) | 5.94  (0.93, 38.07) | **ESWT** | . | . | . | . | . |
| 0.46  (0.16, 1.31) | 1.39  (0.05, 40.98) | 3.71  (0.56, 24.68) | 0.03  (0.00, 0.97) | 0.18  (0.01, 2.79) | 0.32  (0.08, 1.25) | 0.02  (0.00, 0.30) | 0.05  (0.00, 1.14) | 0.07  (0.00, 1.54) | **0.01**  **(0.00, 0.37)** | **EWST_ACB** | . | . | . | . |
| 13.30  (0.76, 232) | 40.47  (0.55, 2998) | 108.29  (4.14, 2835) | 0.81  (0.09, 6.87) | 5.32  (1.37, 20.68) | 9.21  (0.46, 184) | 0.46  (0.21, 0.99) | 1.44  (0.38, 5.48) | 2.14  (0.72, 6.40) | 0.36  (0.06, 2.31) | 29.17  (1.38, 615) | **LIUS** | . | . | . |
| 8.60  (0.47, 57.76) | 26.17  (0.34, 2003) | 70.03  (2.56, 1914) | 0.52  (0.06, 4.74) | 3.44  (0.80, 14.78) | 5.95  (0.28, 125) | 0.30  (0.12, 0.76) | 0.93  (0.22, 3.92) | 1.38  (0.41, 4.68) | 0.23  (0.03, 1.61) | 18.87  (0.85, 416) | 0.65  (0.19, 2.19) | **PROTELOS** | . | . |
| 8.80  (0.62, 126) | 26.78  (0.41, 1741) | 71.65  (3.26, 1575) | 0.53  (0.06, 4.44) | 3.52  (1.49, 8.29) | 6.09  (0.37, 100) | 0.30  (0.15, 0.61) | 0.95  (0.26, 3.49) | 1.42  (0.50, 4.04) | 0.24  (0.04, 1.49) | **19.30**  **(1.11, 337)** | 0.66  (0.23, 1.90) | 1.02  (0.31, 3.33) | **PRP** | . |
| 0.11  (0.00,3.11) | **0.33**  **(0.12, 0.89)** | 0.87  (0.02, 35.96) | **0.01**  **(0.00, 0.78)** | 0.04  (0.00, 2.87) | 0.07  (0.00, 2.42) | **0.00**  **(0.00, 0.29)** | 0.01  (0.00, 1.03) | 0.02  (0.00, 1.43) | **0.00**  **(0.00, 0.31)** | 0.23  (0.01, 8.02) | **0.01**  **(0.00, 0.67)** | 0.01  (0.00, 1.07) | **0.01**  **(0.00, 0.89)** | **PRP_BMA** |

Note: Odds ratios (95% confidence intervals) were calculated. The upper right shows the direct comparison results, and the lower left shows the network comparison results.

Table 2. Pairwise comparisons of the relative effects of interventions on healing time

| **ACB** | 1.42  (-1.16, 4.00) | . | 0.88  (-1.76, 3.51) | . | . | . | 0.65  (-1.97, 3.27) | . | . | 2.58  (-0.30, 5.46) | . |
| --- | --- | --- | --- | --- | --- | --- | --- | --- | --- | --- | --- |
| 1.42  (-1.16, 4.00) | **BMA** | . | . | -1.38  (-4.09, 1.32) | . | . | . | . | . | . | 1.53  (-0.34, 3.40) |
| 2.68  (-1.77, 7.13) | 1.26  (-2.36, 4.89) | **BMP** | . | . | . | . | . | . | -1.56  (-3.44, 0.32) | . | . |
| 0.88  (-1.76, 3.51) | -0.54  (-4.23, 3.15) | -1.80  (-6.98, 3.37) | **BMP_ACB** | . | . | . | . | . | . | . | . |
| 0.03  (-3.70, 3.77) | -1.38  (-4.09, 1.32) | -2.65  (-5.07, -0.23) | -0.84  (-5.42, 3.73) | **CONTROL** | 0.63  (-2.00, 3.26) | 1.02  (-1.95, 3.99) | . | 9.22  (5.35, 13.10) | 1.09  (-0.44, 2.61) | . | . |
| 0.66  (-3.91, 5.23) | -0.75  (-4.52, 3.02) | -2.02  (-5.59, 1.55) | -0.21  (-5.49, 5.06) | 0.63  (-2.00, 3.26) | **CTM** | . | . | . | . | . | . |
| 1.05  (-3.72, 5.82) | -0.36  (-4.38, 3.65) | -1.63  (-5.46, 2.20) | 0.18  ( -5.28, 5.63) | 1.02  (-1.95, 3.99) | 0.39  (-3.58, 4.35) | **EMF** | . | . | . | . | . |
| 0.65  (-1.97, 3.27) | -0.77  (-4.45, 2.91) | -2.03  (-7.20, 3.13) | -0.23  ( -3.94, 3.49) | 0.62  (-3.95, 5.18) | -0.01  (-5.28, 5.25) | -0.40  (-5.85, 5.04) | **EWST_ACB** | . | . | . | . |
| 9.26  (3.87, 14.64) | 7.84  (3.11, 12.57) | 6.57  (2.01, 1.14) | 8.38  (2.38, 14.37) | 9.22  (5.35, 13.10) | 8.59  (3.91, 13.28) | 8.20  (3.32, 13.09) | 8.61  (2.62, 14.60) | **LIUS** | . | . | . |
| 1.12  (-2.92, 5.16) | -0.30  (-3.40, 2.81) | -1.56  (-3.44, 0.32) | 0.24  (-4.58, 5.06) | 1.09  (-0.44, 2.61) | 0.46  (-2.58, 3.50) | 0.07  (-3.27, 3.41) | 0.47  (-4.34, 5.28) | -8.14  (-12.30, -3.97) | **PRP** | . | . |
| 2.58  (-0.30, 5.46) | 1.17  (-2.70, 5.04) | -0.10  (-5.40, 5.21) | 1.71  (-2.19, 5.61) | 2.55  (-2.17, 7.27) | 1.92  (-3.48, 7.32) | 1.53  (-4.04, 7.11) | 1.94  (-1.96, 5.83) | -6.67  (-12.78, -0.56) | 1.46  (-3.49, 6.42) | **PRP_ACB** | . |
| 2.94  (-0.24, 6.13) | 1.53  (-0.34, 3.40) | 0.26  (-3.82, 4.34) | 2.07  (-2.06, 6.20) | 2.91  (-0.37, 6.20) | 2.28  (-1.93, 6.49) | 1.89  (-2.54, 6.32) | 2.30  (-1.83, 6.42) | -6.31  (-11.39, -1.23) | 1.82  (-1.80, 5.45) | 0.36  (-3.93, 4.65) | **PRP_BMA** |

Note: Standard mean differences (95% confidence intervals) were calculated. The upper right shows the direct comparison results, and the lower left shows the network comparison results.

Table 3. Pairwise comparisons of the relative effects of interventions on adverse effects.

| AUTO | 8.14 (1.69, 39.32) | 2.81 (0.11, 74.56) | . | . | . | . |
| --- | --- | --- | --- | --- | --- | --- |
| 8.14 (1.69, 39.32) | BMA+AUTO | . | . | . | . | . |
| 2.81 (0.11, 74.56) | 0.34 (0.01, 13.10) | BMP | . | . | . | 0.33 (0.14, 0.79) |
| 0.97 (0.03, 33.11) | 0.12 (0.00, 5.68) | 0.35 (0.09, 1.28) | CONTROL | 0.08 (0.01, 0.63) | 0.20 (0.06, 0.73) | 0.96 (0.36, 2.59) |
| 0.07 (0.00, 4.52) | 0.01 (0.00, 0.74) | 0.03 (0.00, 0.32) | 0.08 (0.01, 0.63) | EMF | . | . |
| 0.20 (0.00, 8.42) | 0.02 (0.00, 1.42) | 0.07 (0.01, 0.44) | 0.20 (0.06, 0.73) | 2.69 (0.23, 31.96) | ESWT | . |
| 0.93 (0.03, 27.67) | 0.11 (0.00, 4.81) | 0.33 (0.14, 0.79) | 0.96 (0.36, 2.59) | 12.72 (1.22, 132.34) | 4.72 (0.94, 23.58) | PRP |

Note: Odds ratios (95% confidence intervals) were calculated. The upper right shows the direct comparison results, and the lower left shows the network comparison results.

# Subgroup analysis

Long bone fracture nonunion subgroup analysis on healing rate results:

Figure 1. Network plots of the healing rate of therapeutic strategies on nonunion/delayed union in the long bone subgroup network meta-analysis.


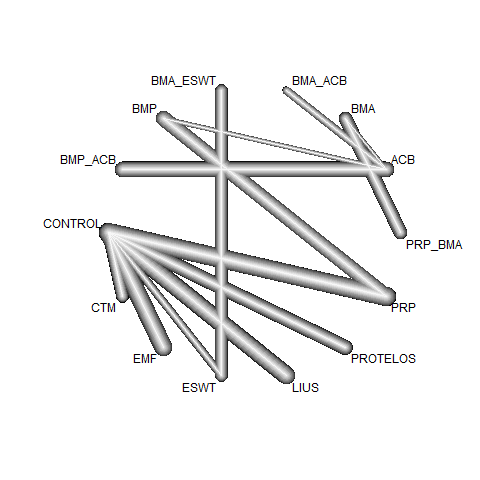


Figure 2. Forest plot of long bone subgroup network meta-analysis comparisons between therapeutic strategies and blank control.


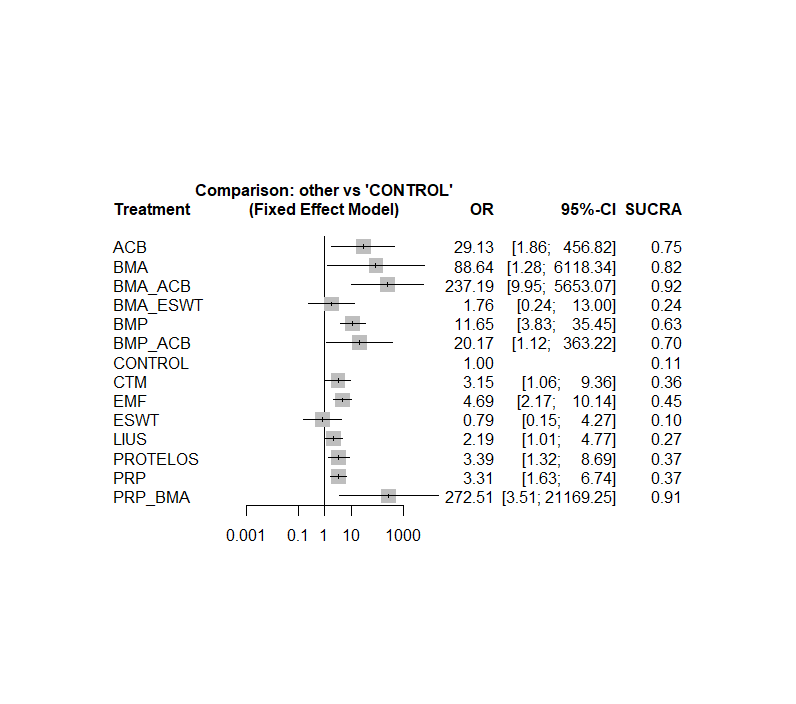


Figure 3. Comparison-adjusted funnel plots of healing rate results in the long bone subgroup network meta-analysis.


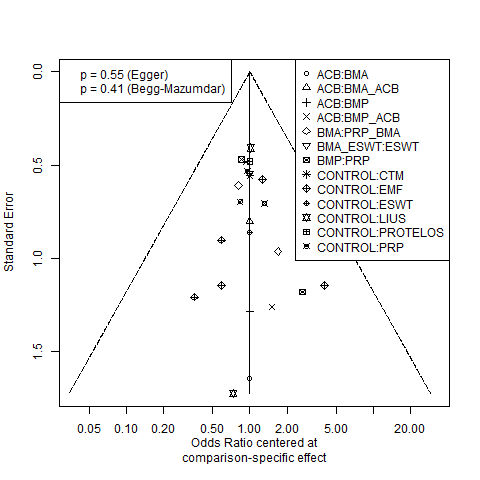


Short bone subgroup of healing rate:

In studies on short bones, only one study reported that EWST_ACB was superior to the ACB strategy in terms of healing rate. In the remaining studies, all fractures recovered in both the intervention and control groups. Subgroup analyses were not performed.

Long bone fracture nonunion subgroup analysis on healing time results:

Figure 4. Network plots of the healing time of therapeutic strategies on nonunion/delayed union in the long bone subgroup network meta-analysis.


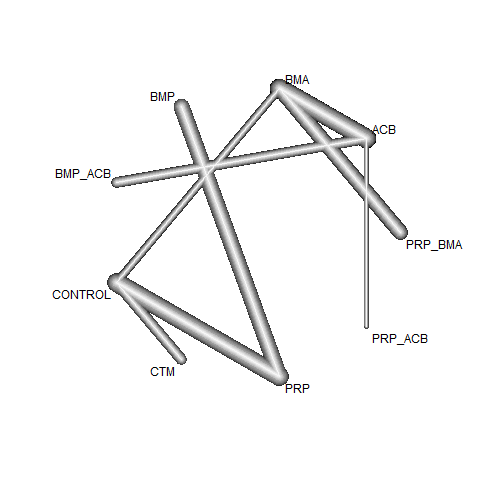


Figure 5. Forest plot of long bone subgroup network meta-analysis comparisons between therapeutic strategies and blank control.


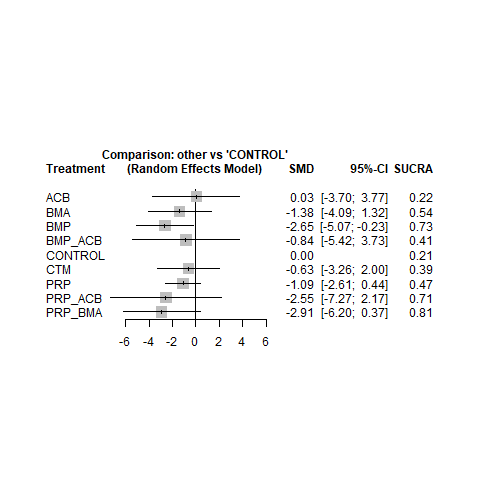


Figure 6. Comparison-adjusted funnel plots of healing rate results in the long bone subgroup network meta-analysis.


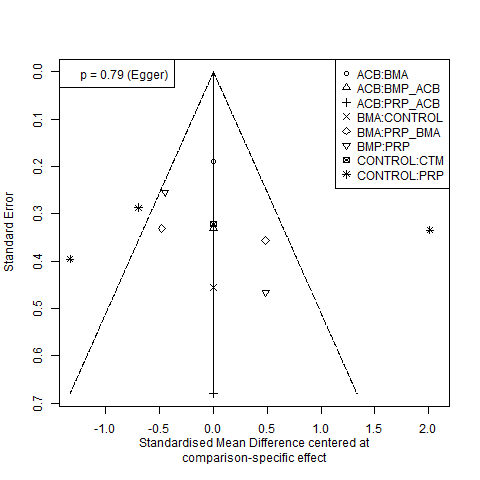


Short bone subgroup analysis of healing time results:

Figure 7. Network plots of the healing time of therapeutic strategies on nonunion/delayed union in the short bone subgroup network meta-analysis.


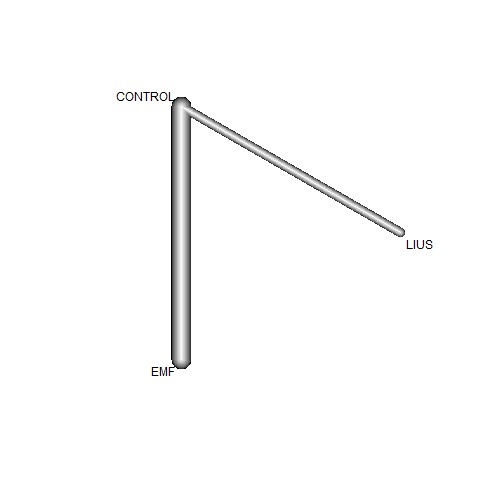


Figure 8. Forest plot of short bone subgroup network meta-analysis comparisons between therapeutic strategies and blank control.


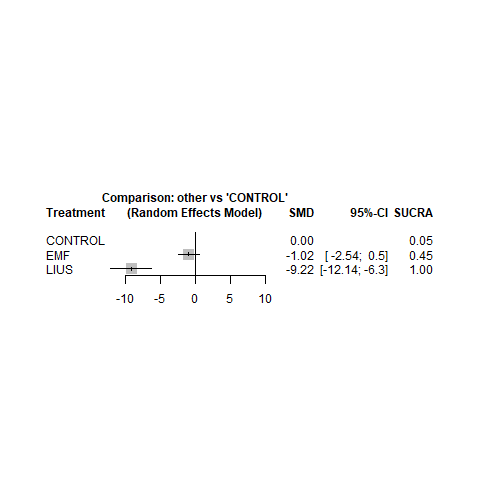


# AE items of each reported study and infection related AE results

Table 1. The adverse effect items from each reported study.

| Study | Adverse effect |
| --- | --- |
| Calori GM 2006 | Reintervention procedure |
| Ghaffarpasand F 2016 | Infection, Mal-union |
| Angelo Cacchio 2009 | Infection, local hematomas, neurapraxia |
| G.M Calori 2008 | Infection, reintervention procedure |
| Hernigou P 2017 | Infection |
| Cook 1999 | Infection |
| Zhao ZC 2017 | Infection |
| Ahmad I 2020 | Drug side effects (nausea, constipation) |
| Friedlaender GE 2001 | Arthralgia, Pain, osteomyelitis, Pyrexia, Vomiting, Edema, Mechanical complication of internal orthopedic device, Hematoma complicating a procedure, Postoperative infection |
| Scott 1994 | Allergic reaction |

Infection related adverse effect subgroup analysis:

Figure 1. Network plots of the infection related adverse effects of therapeutic strategies on nonunion/delayed union


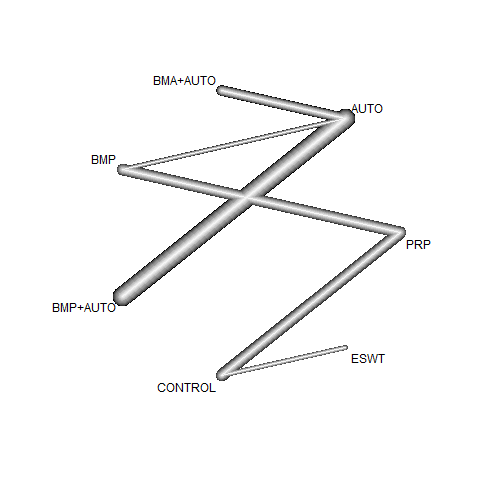


Figure 2. Forest plot of the infection related adverse effect comparisons between therapeutic strategies and the blank control.


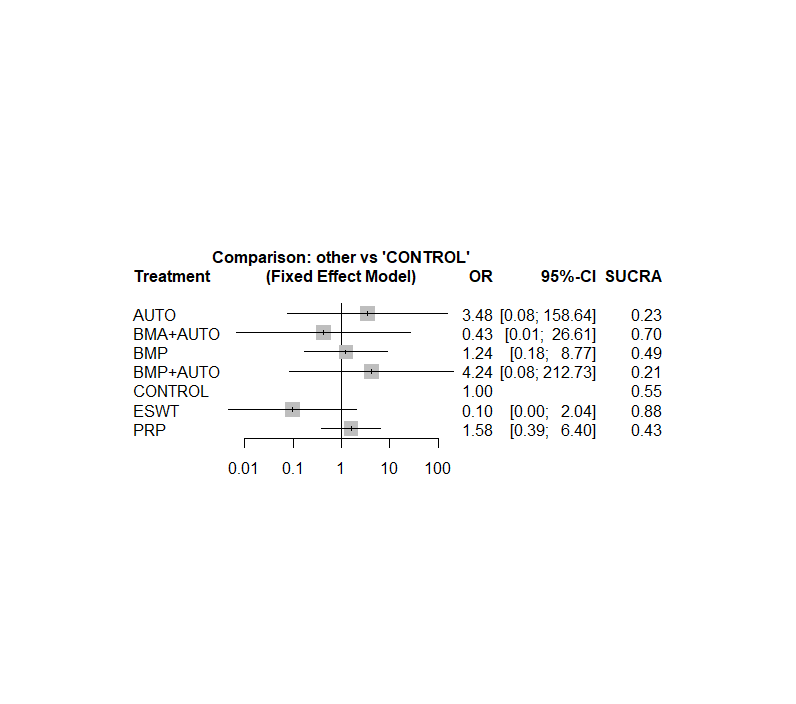

Supplement: Supplementary file 1 — Additional file 1. [file 12891_2022_5407_MOESM1_ESM.docx]
